# Feasibility and acceptability of e-learning to upskill diabetes educators in supporting people experiencing diabetes distress: a pilot randomised controlled trial

**DOI:** 10.1186/s12909-022-03821-w

**Published:** 2022-11-09

**Authors:** Jennifer A. Halliday, Sienna Russell-Green, Virginia Hagger, Eric O, Ann Morris, Jackie Sturt, Jane Speight, Christel Hendrieckx

**Affiliations:** 1 School of Psychology, Deakin University, Geelong, Australia; 2 The Australian Centre for Behavioural Research in Diabetes, Diabetes Victoria, Melbourne, Australia; 3 Institute for Health Transformation, Deakin University, Geelong, Australia; 4 Centre for Quality and Patient Safety Research, School of Nursing and Midwifery, Deakin University, Geelong, Australia; 5 Faculty of Health, Deakin University, Geelong, Australia; 6 AMCON Diabetes Management Service, Warrnambool, Australia; 7 Florence Nightingale Faculty of Nursing, Midwifery and Palliative Care, Kings College London, London, UK

**Keywords:** Diabetes Distress, Diabetes Education, Nurses, Dietitians, Pilot Projects, Randomized Controlled Trial, Feasibility Studies, Education Continuing, Education Distance

## Abstract

**Background:**

Diabetes distress is a commonly experienced negative emotional response to the ongoing burden of diabetes. Holistic diabetes care, including attention to diabetes distress, is recommended in clinical guidelines, yet not routinely implemented. Diabetes health professionals have highlighted lack of training as a barrier to implementation of psychological care. Therefore, we developed an e-learning: ‘Diabetes distress e-learning: A course for diabetes educators’ to address this need. This pilot study aimed to examine the feasibility of evaluating the e-learning in a randomised controlled trial study, the acceptability of the e-learning to credentialled diabetes educators (CDEs); and preliminary evidence of its effect upon CDEs’ diabetes distress-related knowledge, motivation, confidence, behavioural skills, and barriers to implementation.

**Methods:**

A pilot, unblinded, 2-armed, parallel group randomised controlled trial. Participants were recruited during a 4-month timeframe. Eligible participants were CDEs for ≥ 1 year providing care to ≥ 10 adults with type 1 or type 2 diabetes per week. Participants were randomly allocated (1:1 computer automated) to 1 of 2 learning activities: diabetes distress e-learning (intervention) or diabetes distress chapter (active control). They had 4 weeks to access the activity. They completed online surveys at baseline, 2-week and 12-week follow-up.

**Results:**

Seventy-four eligible CDEs (36 intervention, 38 active control) participated. At baseline, recognition of the clinical importance of diabetes distress was high but knowledge and confidence to provide support were low-to-moderate. Engagement with learning activities was high (intervention: 83%; active control: 92%). Fifty-five percent returned at least 1 follow-up survey. All 30 intervention participants who returned the 2-week follow-up survey deemed the e-learning high quality and relevant. Systemic barriers (e.g., financial limitations and access to mental health professionals) to supporting people with diabetes distress were common at baseline and follow-up.

**Conclusions:**

The e-learning was acceptable to CDEs. The study design was feasible but needs modification to improve follow-up survey return. The e-learning showed potential for improving diabetes distress-related knowledge, confidence and asking behaviours, but systemic barriers to implementation remained. Systemic barriers need to be addressed to facilitate implementation of support for diabetes distress in clinical practice. Future larger-scale evaluation of the e-learning is warranted.

**Supplementary Information:**

The online version contains supplementary material available at 10.1186/s12909-022-03821-w.

## Background

The psychological aspects of living with diabetes are intricately intertwined with diabetes management and outcomes [1]. Diabetes distress is a negative emotional response to the ongoing burden of living with and managing diabetes [1], impacting as many as 40% of people with diabetes at any given time [2]. Reducing or preventing diabetes distress is important in its own right [3], doing so may also help to reduce risk of disengagement with diabetes self-care, and from healthcare, thereby reducing sub-optimal biomedical outcomes [4].

People with diabetes expect high quality and person-centred diabetes consultations, including communication about the psychological aspects of diabetes [5, 6]. Diabetes guidelines recommend identification of psychological problems such as diabetes distress [7]. Once identified, diabetes distress can be reduced significantly with support from health professionals through discussion of diabetes-related problem areas [8, 9]. Additionally, psychological approaches (e.g. mindfulness-based therapies) and diabetes education are effective in reducing diabetes distress [10–12].

Unfortunately, psychological problems are often overlooked in routine practice [13, 14]. Many health professionals are aware of the importance of psychological care in diabetes, yet they lack the resources, training, and confidence to assess for, communicate about, and address such problems [15, 16]. Training courses exist to upskill health professionals in diabetes counselling [17], but face-to-face trainings have limited reach (e.g. due to geography and capacity) [18] and their outcomes (e.g. knowledge, communication skills, behaviour change) are rarely evaluated. Conversely, research projects designed to develop and evaluate health professionals’ consultation skills training [19–21] are often constrained by funding limitations impeding ongoing implementation of the training. Textbooks also exist, yet, often, they do not include practical elements to facilitate skill development and are hidden behind paywalls [22]. To overcome such limitations, the ‘Diabetes and Emotional Health’ practical guide was developed in Australia [1, 22], to give health professionals free-to-access, evidence-based, step-by-step information and tools required for providing diabetes-related psychological support. The resource includes a chapter on diabetes distress. It is well received by health professionals [22] and adapted for the UK, USA and Denmark [23–25]. However, evaluation suggested that some health professionals would prefer the resource in a more interactive training format, to help them enhance their confidence and consultation skills [22]. In response, we developed diabetes distress e-learning, to supplement the practical guide [26].

The aim of this study was to examine the 1) feasibility of evaluating the e-learning in a randomised controlled trial study design 2) acceptability of the e-learning to credentialled diabetes educators (CDEs^1^), 3) preliminary evidence of effect of the e-learning upon CDEs’ diabetes distress-related knowledge, motivation, confidence, behavioural skills, and barriers to implementation.

## Methods

**Fig. 1:**
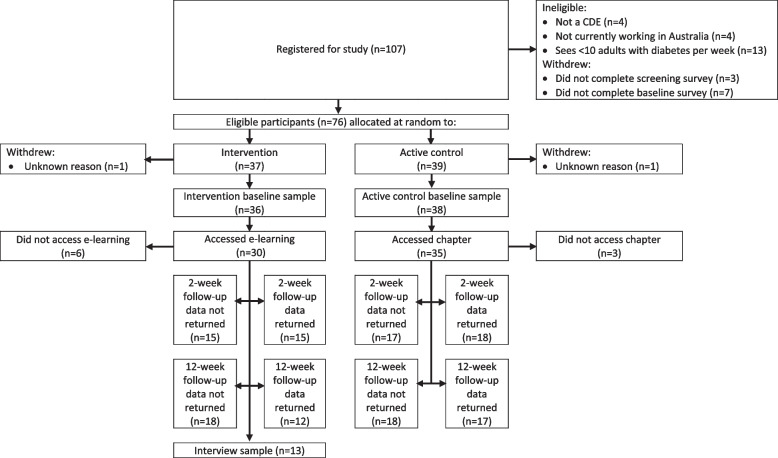
Study design flowchart with participation and data return summary

**Fig. 2:**
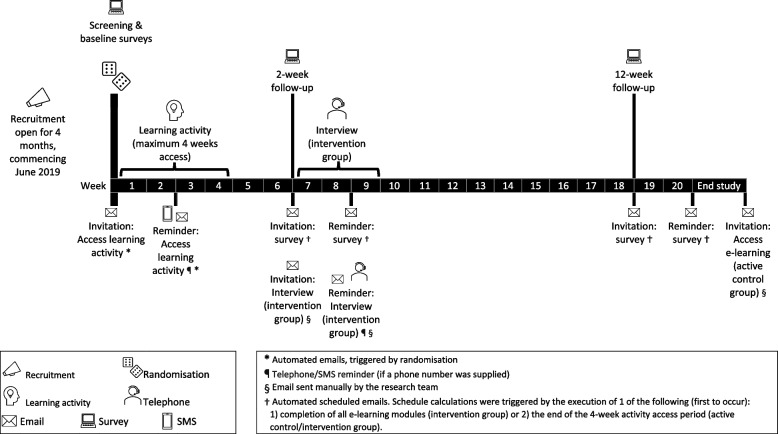
Timeline of the randomised controlled trial

We conducted a pilot, unblinded, 2-armed, parallel group, randomised controlled trial of ‘Diabetes distress e-learning: A course for diabetes educators’ (Figs. 1 and 2). In June 2019 we commenced promoting the study to CDEs in our existing database who had expressed interest in research participation opportunities, and via health professional meetings/conferences and relevant professional organisations. The promotional materials directed prospective participants to a website for self-registration of interest to participate in the study. The prospective participants were screened for eligibility via a short online Qualtrics survey. The eligibility criteria were: 1) qualified and currently working as a CDE in Australia for ≥ 1 year, 2) direct involvement in the clinical care of ≥ 10 adults with type 1 or type 2 diabetes per week, and 3) desktop or laptop computer access with an internet connection.

Following online completion of baseline survey questions (see Measures), eligible participants were randomly allocated (automated computer-generated, 1:1 ratio) to the intervention group (diabetes distress e-learning) or active control group (diabetes distress chapter). The computer-generated randomization was facilitated independently by the platform developer. Participants were instructed how to access their allocated learning activity at the end of the survey and via email and had 4 weeks to access the activity.

At 2- and 12-weeks follow-up, all participants were invited to complete online (Qualtrics) surveys to assess immediate and longer-term outcomes (e.g., implementation), respectively. To minimise missing data, all fixed choice survey questions (at all timepoints) were set as ‘forced response’. The intervention group also provided feedback about acceptability and implementation of the e-learning in their 2-week follow-up survey and during a semi-structured telephone interview. The interview methods and findings will be reported elsewhere.

Participants completing the study were eligible to receive a certificate, which could be used for professional credentialling purposes, and a hard copy of ‘Diabetes and Emotional Health’. Interview participants were offered entry to a prize draw ($200 voucher). No incentives were offered for survey return. The active control group were offered equivalent (4 weeks) access to the e-learning at the end of the study.

### Learning activities

Active control participants were emailed a hyperlink to the diabetes distress chapter of ‘Diabetes and Emotional Health’ [1, 22] and instructed to read it. The chapter follows a 7As model (AWARE, ASK, ASSESS, ADVISE, ASSIST, ASSIGN, and ARRANGE) to guide learners in a stepwise process for identifying and addressing diabetes distress. The chapter was developed in consultation with stakeholders and has foundations in person-centred care [22]. It provides comprehensive evidence-based information about diabetes distress, including signs to look for, suggested open-ended questions, and case studies. It also includes a copy of the Problem Areas in Diabetes scale (PAID; a validated diabetes distress questionnaire) with guidance about scoring, interpretation, and discussion. It is freely available online (www.ndss.com.au). A summary of the chapter content is provided in Supplement 1.

Intervention participants were emailed a hyperlink to the e-learning and instructed to access it. The e-learning contains similar information to the diabetes distress chapter. The key difference is that the e-learning is interactive, with greater emphasis on skills development, informed by educational and behavioural theoretical models. For example, it includes learning objectives, videos demonstrating consultation skills, and activities (e.g., opportunities to check understanding, practice skills, self-reflect, and plan actions). It was developed using intervention mapping, a comprehensive best-practice framework for informing the planning, development and evaluation of an intervention [26]. Additional details about the platform, e-learning content, and evaluation plan (e.g. pre-defined study design and outcomes of interest) are published elsewhere [26].

### Sample size and ‘stop/go’ criteria

While pilot studies do not require a sample size calculation, we aimed for *N*= 50, anticipating 20% attrition, allowing for a minimum of 10 participants per arm [27]. We monitored participant recruitment, engagement with the intervention (module completion) and follow-up data return to ensure sufficient data were collected. No ‘stop/go’ criteria (other than achievement of sample size) or interim analyses were planned or implemented.

### Deviations from protocol

The study promotion and recruitment period was extended from 2 months, to about 4 months, due to slower than expected uptake and low follow-up survey response rates. We added reminders to participate, for both the learning activity and follow-up data collection. We extended the learning activity access timeframe from 2 to 4 weeks for all participants, following early participant feedback that 2 weeks was too short due given busy clinical schedules. We recruited more than the planned 50 participants due to higher than anticipated attrition. Due to the lengthening of timeframes for recruitment and activity access, we completed data collection in early in 2020, which was later than initially planned.

### Outcomes

Study design feasibility: Recruitment speed/success, exclusion criteria suitability (rates of and reasons for exclusion), engagement (participant withdrawals and learning activity access), attrition (follow-up data return), and suitability of study-specific measures (floor/ceiling effects, scale reliability of the confidence and importance survey measures).

Intervention acceptability: Intervention completion times, number of modules accessed, user ratings of intervention quality and acceptability (see Survey measures).

Preliminary evidence of effects: CDEs’ diabetes distress-related knowledge, motivation, confidence, behavioural skills, and barriers to implementation (see Survey measures).

#### Survey measures

Informed by an intervention mapping process (described elsewhere [26]), study-specific survey questions were developed to measure intervention quality and acceptability and preliminary evidence of effects:

- *Quality, acceptability, and future applications* (2-week follow-up; intervention group only)*:* Twenty-six items assessed user experiences including the quality and relevance of the content, suitability and ease-of-use of the platform, and future applications. Of these 25, were assessed on a 4-point scale (’strongly disagree’ to ‘strongly agree’) and 1 question had a ‘yes/no’ response format. Four additional questions assessed the appropriateness of the time required, difficulty level and technical problems.
- *Knowledge* (all timepoints; all participants): Twelve questions about their knowledge of diabetes distress (response options: true/false/I don’t know). Each e-learning module was represented by 1–2 questions. Correct responses were summed (possible scores: 0–12), with higher scores indicating greater knowledge of diabetes distress.
- *Perceived importance of providing support* (all timepoints; all participants): Three scales with items describing clinical behaviours/actions: identify diabetes distress (5 items), assist with diabetes distress (3 items), and refer for diabetes distress (3 items) (Supplement 2). For each item, participants rated the importance of each action on a 4-point scale (0 = ‘not at all important’ to 3 = ‘very important’). For each of the 3 scales, the item scores were summed to form a total score.
- *Confidence to provide support* (all timepoints; all participants): The same scales/actions/scoring were repeated as the ‘importance’ items, but participants were asked to indicate their confidence to implement each action (0 = ‘not at all confident’ to 3 = ‘very confident’).
- *Behavioural skills* (all timepoints; all participants): One question enquired about the proportion of adults with type 1 or type 2 diabetes the CDE asked “how they feel about living with and managing diabetes” in the past 2 weeks (i.e., Ask about diabetes distress). Two similarly worded question enquired about assessment of diabetes distress (i.e., the proportion they invited to complete a diabetes distress questionnaire, e.g., PAID or Diabetes Distress Scale) and assessment of mental health (i.e., the proportion they invited to complete another psychological questionnaire, e.g., Patient Health Questionnaire 9, Kessler Psychological Distress Scale 10, or World Health Organisation Wellbeing Index Five). All items were rated on a 4-point scale (0 = ‘none of them’ to 3 = ‘all of them’).
- *Workplace and systemic barriers* (all timepoints; all participants): Participants rated the extent to which 18 potential barriers/enablers affect them in providing support for diabetes distress. The items (e.g., workplace set-up and policies, access to tools and resources, mental health referral options, funding and remuneration) were each rated on a 5-point scale (-2 = ‘hinders me a lot’ to 2 = ‘helps me a lot’).

In addition, at baseline, participants completed eleven items about their demographic and professional characteristics (e.g., age, gender, profession, workplace geography and setting). They also reported on relevant *Prior relevant professional development* via 2 questions*:* whether they had previously: a) read *‘Diabetes and Emotional Health’* (response options: yes—in part, yes – in full, no); b) participated (in past 5 years) in consultation skills training (response options: yes/no). We listed various training examples relevant to diabetes, mental health, communication, and counselling skills (e.g., Dose Adjustment For Normal Eating (DAFNE) or Diabetes Education and Self-Management for Ongoing and Newly Diagnosed (DESMOND) facilitation, acceptance commitment therapy, motivational interviewing, mental health assessment or counselling, consultation skills masterclass) as prompts.

### Analysis

Descriptive statistics were used to describe participant characteristics, e-learning acceptability, and barriers to support. The internal consistency reliability of the importance and confidence scales was evaluated using the baseline survey data (Cronbach’s alpha, α > 0.7 was deemed acceptable). Preliminary effect of the e-learning program was explored by comparing within group changes over time using Wilcoxon Signed Rank Tests and effect sizes (*r* values). Most analyses were conducted in SPSS (28.0.0.0, 190). Wilcoxon Signed Rank Tests effect sizes were calculated in Microsoft Excel (2203, 16.0.15028.20178). The e-learning time commitment was calculated in Microsoft Excel, informed by website analytics.

## Results

### Participants’ characteristics, at baseline

Most participants were women (97%) and born in Australia (73%). Their mean age was 51 years. Many had a nursing background (84%), consulted primarily in English (88%), and had a minimum of 10 years diabetes-related work experience (60%). Many worked in metropolitan (43%) or regional (42%) geographical settings, fewer (15%) worked rurally. They worked across a range of workplace settings, most commonly in community health (28%), public hospitals (22%), general practice (14%) or private practice (14%). Most worked as part of a multidisciplinary team (85%). Most had participated in other mental health or communication-related training in the past 5 years (86%).

### Knowledge, importance, confidence, and behaviour, at baseline

Most participants perceived providing support for diabetes distress as important, but they had varying levels of knowledge about and confidence to provide such support (Table 1). Asking about and assessing for diabetes distress was uncommon in the 2 weeks prior to the study commencement.

**Table 1:** Participant characteristics at baseline, by group allocation

Characteristics	Intervention (*N* = 36)	Active Control (*N* = 38)
	**N (%) or Median [range]**	
Profession: CDE with background in:		
Nursing (including midwives and nurse practitioners)	32 (88.9)	30 (79.0)
Dietetics	3 (8.3)	7 (18.4)
Pharmacy	1 (2.8)	1 (2.6)
Diabetes consultation experience (years)		
1–5	5 (13.9)	5 (13.2)
6–10	11 (30.6)	9 (23.7)
> 10	20 (55.6)	24 (63.2)
Primary workplace geography		
Metropolitan	15 (41.7)	17 (44.7)
Regional	17 (47.2)	14 (36.8)
Rural	4 (11.1)	7 (18.4)
Primary workplace setting		
Community health centre	11 (30.6)	10 (26.3)
General practice (primary care)	6 (16.7)	4 (10.5)
Non-for-profit or non-government organisation	5 (13.9)	4 (10.5)
Private hospital	3 (8.3)	1 (2.6)
Private practice	5 (13.9)	5 (13.2)
Public hospital	6 (16.7)	10 (26.3)
Other	0 (0.0)	4 (10.5)
Work colleagues		
Multidisciplinary without mental health professional	16 (44.4)	22 (57.9)
Multidisciplinary with mental health professional	14 (38.9)	11 (29.0)
Work alone or in single discipline group	6 (16.7)	5 (13.2)
Age (years)	53.5 [26.0─65.0]	50.5 [29.0─60.0]
Gender: Female	34 (94.4)	38 (100.0)
Country of birth: Australia	26 (72.2)	28 (73.7)
Primary language during consultations: English only	34 (94.4)	31 (81.6)
Prior relevant professional development activities		
Read diabetes distress chapter of *‘Diabetes and Emotional Health*’: Yes, in full or partially	23 (63.9)	26 (68.4)
Consultation skills training (past 5 years): Yes	30 (83.3)	34 (89.5)
Correct diabetes distress knowledge questions (out of 12)	7.0 [4.0─10.0]	8.0 [4.0─11.0]
Importance scales		
Identify diabetes distress (5 items, total score: 0–15)	12.5 [4.0─15.0]	12.0 [7.0─15.0]
Assist with diabetes distress (3 items, total score 0–9)	9.0 [3.0─9.0]	9.0 [6.0─9.0]
Refer diabetes distress (3 items, total score 0–9)	9.0 [3.0─9.0]	8.50 [5.0─9.0]
Confidence scales		
Identify diabetes distress (5 items, total score 0–15)	7.0 [0.0─15.0]	6.0 [0.0─14.0]
Assist with diabetes distress (3 items, total score 0–9)	4.0 [0.0─9.0]	3.0 [0.0─9.0]
Refer diabetes distress (3 items, total score 0–9)	5.0 [0.0─9.0]	4.5 [0.0─9.0]
Behavioural skills		
Ask about diabetes distress (1 item, scored 0–3)	1.0 [0.0─3.0]	1.00 [1.0─3.0]
Assess diabetes distress (1 item, scored 0–3)	0.0 [0.0─3.0]	0.0 [0.0─3.0]
Assess mental health (1 item, scored 0–3)	0.0 [0.0─2.0]	0.0 [0.0─2.0]

### Barriers and enablers to support for diabetes distress, at baseline

**Fig. 3:**
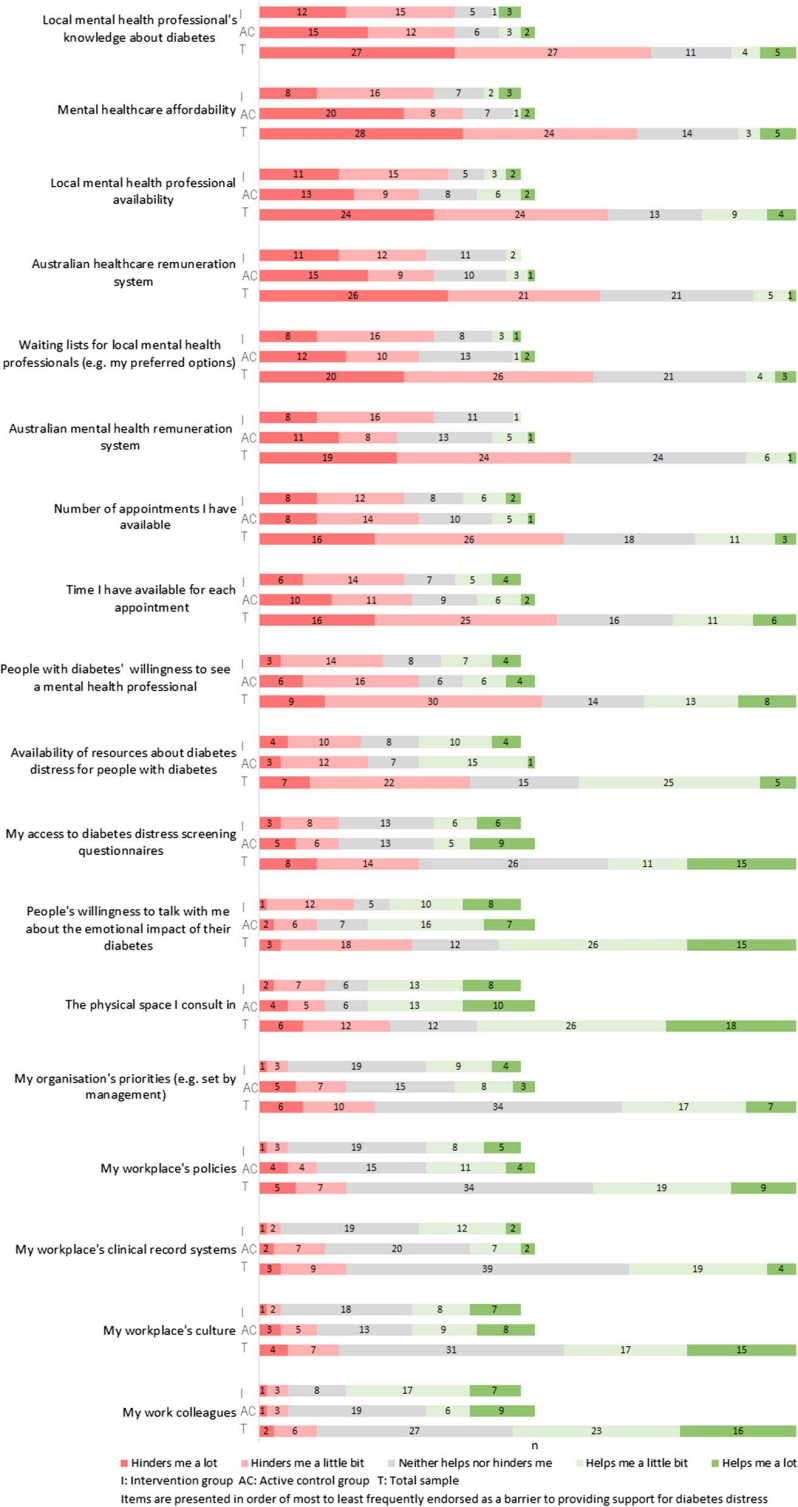
Workplace and systemic barriers and enablers to support for diabetes distress at baseline

At baseline, the most frequently cited barriers to providing support for diabetes distress related to referral and funding/remuneration. For example, mental health professionals’ (lack of) knowledge about diabetes (73% of participants), affordability of mental healthcare (70%), availability of mental health professionals (65%) and waiting lists (62%), and the remuneration system for healthcare (64%) and mental healthcare (58%) (Fig. 3). The most frequently selected enablers of providing support for diabetes distress were: consulting spaces (59%), the willingness of people with diabetes to talk about the emotional aspects of diabetes (55%), and work colleagues (53%).

### Study design feasibility

#### Exclusions, withdrawals, and randomisation

One fifth of registrants (*n* = 21/107) did not meet the eligibility criteria and were excluded; many of whom did not meet the criteria for consulting with ≥ 10 people with diabetes per week (*n* = 13/21). Seventy-eight of the 86 eligible CDEs (91%) completed the baseline questionnaire and were randomly allocated to the intervention or active control group. One participant from each arm withdrew, thus their data were removed, leaving 36 intervention group and 38 active control group participants (Fig. 1).

#### Scale reliability

The 3 importance and 3 confidence scales all had acceptable reliability (α > 0.7; Supplement 2).

#### Learning activity access

The allocated learning activity was accessed by 83% (*n* = 30/36) of intervention and 92% (*n* = 35/38) of active control participants (Fig. 1). The median number of e-learning modules accessed was 6 (range: 0–7), with 17 participants (47%) accessing all 7 modules.

#### Attrition

Loss to follow-up was higher than anticipated; of 74 the consenting and eligible participants, 45% did not return either follow-up surveys, 27% returned 1 follow-up survey, and 28% returned both follow-up surveys. Follow-up survey data were returned by 53% of the intervention group and 58% of the active control group. Most intervention group participants who returned follow-up survey data accessed all 7 modules (14/19; 74%), whilst the rest accessed 1 to 6 modules. Conversely, among intervention participants who did not return follow-up data: one third did not access the intervention (6/17), one third accessed 2 to 3 modules (6/17), and one third accessed 6 to 7 modules (5/17).

### Intervention acceptability

#### Time commitment and difficulty

**Fig. 4:**
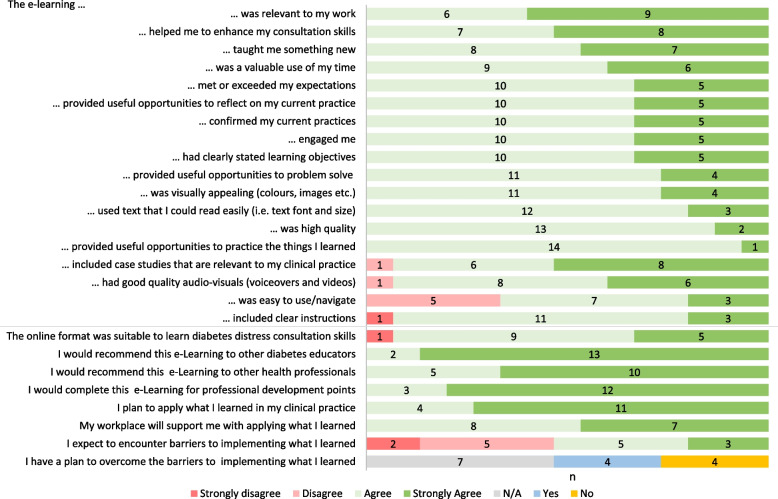
Participant ratings of the diabetes distress e-learning

The average time spent that intervention group participants spent on the e-learning was about 7 h (42–73 min per module). This time commitment was deemed ‘about right’ by 87% (*n* = 13/15; Fig. 4) of participants, whilst 1 participant indicated it was ‘somewhat too long’ and 1 ‘too short’. Ninety-three percent (*n* = 14/15) agreed/strongly agreed that they could complete the e-learning at a pace that suited them, 1 participant disagreed. The difficulty level was ‘about right’ for 93% (*n* = 14/15) of participants, 1 rated it ‘too difficult.’

#### Content relevance, quality, and future applications

All intervention group participants agreed/strongly agreed that the e-learning was relevant to their clinical practice, a valuable use of their time, engaging, visually appealing and high quality (Fig. 4). All participants planned to apply what they learned to their clinical practice, but half (*n* = 8/15) expected barriers to implementation and a quarter (*n* = 4/15) did not have a plan to overcome them.

#### Platform suitability and quality

Most intervention group participants agreed/strongly agreed (*n* = 14/15; 93%) that the online e-learning format was ‘suitable to learn consultation skills for diabetes distress’. Many agreed/strongly agreed (*n* = 10/15; 67%) that the e-learning was ‘easy to use/navigate’. Forty percent (*n* = 6/15; Fig. 4) experienced technical difficulties.

### Preliminary evidence of effect

#### Change in knowledge, importance, confidence, and behaviour scores

At 2-week follow-up, compared to baseline, significantly higher knowledge about diabetes distress and confidence to identify, assist with, and refer people with diabetes distress were observed in the intervention and active control groups (moderate-to-high effect: *r* = 0.42 to 0.63, all *p* ≤ 0.01; Table 2). Higher perceived importance to identify diabetes distress was observed in the active control group (*r* = 0.53, *p* < 0.01).

**Table 2:** Change between Baseline and 2-week follow-up within groups

Construct	Group	Baseline: Median [range]	2- week follow-up: Median [range]	*T*	*z*	*p*	*r*
Diabetes distress knowledge	Intervention	7.00 [4.00—9.00]	11.00 [7.00—12.00]	95.50	-3.43	** < 0.001**	0.63
	Active control	8.00 [6.00—11.00]	10.00 [6.00—12.00]	120.00	-2.72	** < 0.01**	0.45
Importance: Identify diabetes distress	Intervention	13.00 [7.00—15.00]	12.00 [8.00—15.00]	45.50	0.00	1.00	
	Active control	12.00 [7.00—15.00]	14.00 [9.00—15.00]	158.50	-3.21	** < 0.001**	0.53
Importance: Assist with diabetes distress	Intervention	9.00 [6.00—9.00]	9.00 [6.00—9.00]	6.00	-0.38	1.00	
	Active control	9.00 [3.00—9.00]	9.00 [6.00—9.00]	15.50	-1.06	0.31	
Importance: Refer diabetes distress	Intervention	9.00 [3.00—9.00]	9.00 [6.00—9.00]	14.50	-0.09	1.00	
	Active control	9.00 [5.00—9.00]	9.00 [6.00—9.00]	28.00	-0.66	0.58	
Confidence: Identify diabetes distress	Intervention	5.00 [0.00—11.00]	10.00 [4.00—14.00]	103.00	-3.19	** < 0.001**	0.58
	Active control	6.50 [0.00—10.00]	9.00 [2.00—15.00]	129.00	-3.17	** < 0.001**	0.53
Confidence: Assist with diabetes distress	Intervention	3.00 [0.00—6.00]	6.00 [2.00—9.00]	91.00	-3.21	** < 0.001**	0.59
	Active control	3.50 [0.00—8.00]	5.00 [2.00—9.00]	94.50	-2.67	** < 0.01**	0.44
Confidence: Diabetes distress referral	Intervention	3.00 [1.00—8.00]	4.00 [2.00—9.00]	80.50	-2.48	**0.01**	0.45
	Active control	5.00 [0.00—9.00]	5.00 [2.00—9.00]	81.00	-2.50	**0.01**	0.42
Ask about diabetes distress	Intervention	1.00 [1 .00—3.00]	2.00 [1.00—3.00]	21.00	-1.27	0.36	
	Active control	1.00 [1.00—3.00]	2.00 [1.00—3.00]	14.00	-0.82	0.69	
Assess diabetes distress	Intervention	0.00 [0.00—1.00]	0.00 [0.00—2.00]	6.00	-1.63	0.25	
	Active control	0.00 [0.00—2.00]	0.05 [0.00—2.00]	16.00	-0.38	1.00	
Assess mental health	Intervention	0.00 [0.00—2.00]	0.00 [0.00—2.00]	2.00	-0.58	1.00	
	Active control	0.00 [0.00—2.00]	0.00 [0.00—3.00]	1.50	0.00	1.00	

Intervention N = 15, Active control N = 18

Wilcoxon Signed Ranks tests

**Bolded** *p* values indicate significant differences (*p* < 0.05) within groups

At 12-week follow-up, compared to baseline, significantly higher knowledge about diabetes distress, and confidence to identify, assist with, and refer people with diabetes distress were observed in the intervention group (moderate-to-high effect: *r* = 0.46 to 0.59, all *p* ≤ 0.02; Table 3). Intervention participants also asked a higher proportion of adults with diabetes about diabetes distress compared to baseline (*r* = 0.50, *p* = 0.03; Table 3). Significantly higher knowledge about diabetes distress; confidence to identify and assist with diabetes distress; and perceived importance to identify, assist with, and refer people with diabetes distress were observed in the active control group (moderate-to-high effect: *r* = 0.41 to 0.55, all *p* ≤ 0.02).

**Table 3:** Change between Baseline and 12-week follow-up within groups

Construct	Group	Baseline: Median [range]	12- week follow-up: Median [range]	*T*	*z*	*p*	*r*
Diabetes distress knowledge	Intervention	7.00 [4.00—9.00]	8.00 [7.00—11.00]	60.00	-2.42	**0.02**	0.49
	Active control	8.00 [4.00—11.00]	10.00 [7.00—12.00]	93.50	-2.60	** < 0.01**	0.45
Importance: Identify diabetes distress	Intervention	12.50 [7.00—15.00]	12.00 [9.00—15.00]	20.00	-0.30	0.79	
	Active control	12.00 [8.00—15.00]	13.00 [9.00—15.00]	81.00	-2.51	**0.01**	0.43
Importance: Assist with diabetes distress	Intervention	9.00 [3.00—9.00]	8.50 [5.00—9.00]	11.00	-0.11	0.81	
	Active control	9.00 [6.00—9.00]	9.00 [8.00—9.00]	28.00	-2.43	**0.02**	0.42
Importance: Diabetes distress referral	Intervention	8.50 [3.00—9.00]	9.00 [6.00—9.00]	18.00	-0.70	0.53	
	Active control	8.00 [5.00—9.00]	9.00 [7.00—9.00]	51.00	-2.41	**0.02**	0.41
Confidence: Identify diabetes distress	Intervention	6.00 [0.00—11.00]	9.00 [3.00—15.00]	75.00	-2.88	** < 0.001**	0.59
	Active control	6.00 [2.00—10.00]	9.00 [5.00—15.00]	130.00	-3.22	** < 0.002**	0.55
Confidence: Assist with diabetes distress	Intervention	3.00 [0.00—6.00]	6.00 [1.00—9.00]	49.50	-2.27	**0.02**	0.46
	Active control	3.00 [0.00—6.00]	6.00 [2.00—9.00]	130.00	-3.23	** < 0.001**	0.55
Confidence: Diabetes distress referral	Intervention	3.00 [1.00—8.00]	6.00 [2.00—9.00]	52.50	-2.57	**0.01**	0.52
	Active control	5.00 [2.00—7.00]	6.00 [1.00—9.00]	82.50	-1.91	0.06	
Ask about diabetes distress	Intervention	1.00 [1.00—2.00]	2.00 [1.00—3.00]	21.00	-2.45	**0.03**	0.50
	Active control	1.00 [1.00—2.00]	2.00 [1.00—2.00]	10.00	-2.00	0.13	
Assess diabetes distress	Intervention	0.00 [0.00—1.00]	1.00 [0.00—2.00]	31.50	-2.12	0.07	
	Active control	0.00 [0.00—2.00]	0.00 [0.00—2.00]	25.00	-0.33	1.00	
Assess mental health	Intervention	0.00 [0.00—2.00]	0.00 [0.00—1.00]	9.00	-0.45	1.00	
	Active control	0.00 [0.00—2.00]	0.00 [0.00—3.00]	1.50	0.00	1.00	

Intervention *N* = 12. Active control *N* = 17

Wilcoxon Signed Ranks tests

**Bolded** *p* values indicate significant differences (*p* < 0.05) within groups

#### Change in barriers to support for diabetes distress in clinical practice

At 2-week follow-up, compared to baseline, a significant reduction of moderate effect was observed in the intervention group for 2 barriers: workplace policies (moderate effect: *r* = 0.41, *p* < 0.03) and access to screening questionnaires (high effect: *r* = 0.52, *p* < 0.01; Supplement 3).

At 12-week follow-up, compared to baseline, a significant improvement of high effect was observed in the intervention group regarding 2 barriers: access to screening questionnaires (high effect: *r* = 0.52, *p* < 0.01) and access to diabetes distress-related resources for people with diabetes (high effect: *r* = 0.58, *p* < 0.01; Supplement 4).

No significant changes from baseline scores were observed in the active control group at either follow-up.

## Discussion

The study findings indicate that the e-learning is worthy of further evaluation in a larger trial, pending modification to improve feasibility of the study design. The diabetes distress e-learning was acceptable to CDEs, and the e-learning showed promise for improving CDE’s knowledge, confidence, and clinical behaviours related to providing support for diabetes distress.

Regarding study design feasibility, we were able to achieve the intended outcomes of the study, such as recruiting the planned sample size. However, we had to make some modifications to the study design to achieve this. For instance, recruitment to the study was successful once the recruitment timeframe was lengthened. Initial study engagement was high (86%): of the 76 eligible participants, only 2 withdrew and 9 opted not to access their allocated activity. Follow-up survey return was lower than expected in both groups, with slightly higher follow-up response rates in the active control group. A possible explanation is that the active control group had an additional incentive to stay engaged, to gain access to the e-learning at the conclusion of the study. The addition of reminders (to complete the allocated activity/follow-up surveys) likely helped to improve participant follow-up response rates, yet other strategies are needed. Time is a known barrier to health professional participation in research, as they often have competing priorities that take precedence, thus, loss to follow-up is common [28]. We were able to explore time as a barrier to participation during the qualitative interviews and will report the findings in a subsequent publication. Future evaluation of the e-learning needs to consider ways to reduce participant time-burden (e.g. fewer timepoints and/or shorter surveys) [28]. Ways to boost participant recruitment and retention should also be explored, including financial incentives/remuneration for time, mixed-mode multiple reminders, and gaining support from gatekeepers (e.g. management) [28–31]. Alternative study designs, such as hybrid implementation trials (e.g. embedded within workplaces), could also be considered for future evaluations [32]. Additionally, broadening the study eligibility criteria in future evaluations will enable more CDEs to participate. Specifically, 1 exclusion criterion of the current pilot study (CDE consults with ≥ 10 adults with type 1 or type 2 diabetes weekly) was intentionally stringent, to ensure the participants had sufficient opportunity in their clinical practice for implementation of the e-learning. Resultingly, it led to disproportionate exclusions compared to the other criteria; such a ‘tight’ criterion may not be necessary in future larger-scale evaluations of the e-learning. The study-specific measures appeared to work as intended, with acceptable reliability and a range of item responses, except for the ‘importance’ scale, which showed a ceiling effect at all 3 timepoints. This is unsurprising as the participants were volunteers wanting to ‘upskill’ in diabetes distress. Topic interest is a known facilitator to research participation among health professionals [29].

The e-learning was acceptable to the intervention group participants who accessed it. They perceived the online format as suitable for learning about diabetes distress and 93% considered the time commitment ‘about right’ or ‘too short’. They viewed the e-learning content as high quality, relevant, engaging, and helpful for enhancing consultation skills. A few participants experienced problems related to platform navigation and technical difficulties. Further details of these problems and suggestions for improvement were explored qualitatively (outside scope of this paper); they will be resolved in future versions of the e-learning. Overall, the satisfactory acceptability of the e-learning demonstrates that it shows promise for future uptake by CDE’s. About a third of intervention participants who accessed 1 or more modules (11/30) did not return follow-up surveys; thus their experiences of the intervention are unknown to us.

Based on the findings of this pilot study, both the e-learning and the chapter show potential utility for increasing CDEs knowledge and confidence to provide support for diabetes distress in their clinical practice. Offering both complementary resources could enable CDEs to select the resource most suited to their preferences and needs [26]. However, there were some noteworthy instances where significant improvements were observed only in the e-learning group. One likely explanation is the additional application of theory-informed components, such as behaviour change techniques (BCTs), within the e-learning. CDE’s confidence to refer improved only in the intervention group at 12-week follow-up. Both the chapter and e-learning include similar information about referrals, such as suggestions about when and how to make a mental health referral. However, the e-learning additionally utilised BCTs. For instance, the e-learning included a video of an experienced CDE describing her experience of overcoming barriers to diabetes distress-related mental health referral (BCTs: ‘Credible source’, ‘Instruction on how to perform the behaviour’, ‘Demonstration of the behaviour’) [33]. In related activities, the participants 1) identified their own barriers and planned strategies for overcoming them and 2) planned feasible strategies to improve their communication with mental health professionals (BCT: ‘Problem solving’) [33]. Additionally, the intervention group reported asking more people about diabetes distress during their consultations, at 12-week follow-up. Both the chapter and e-learning included similar related practical information (e.g., suggestions for open-ended questions and responses for talking about diabetes distress) but the e-learning additionally used BCTs. For instance, it included video demonstrations of an experienced CDE integrating open-ended questions/responses into a clinical conversation (BCTs: ‘Credible source’*, ‘*Instruction on how to perform the behaviour’, and ‘Demonstration of the behaviour’) [33]. In related activities, the CDEs practiced writing open-ended questions/responses in response to various case studies (BCT: ‘Behavioural practice/rehearsal’), received feedback (BCT: ‘Feedback on behaviour’), self-reflected on their strengths (BCT: ‘Valued self-identity’), and made plans for integrating conversations about diabetes distress into their clinical practice (BCT: ‘Action planning’) [33].

Notably, the aforementioned improvement in frequency of asking about diabetes distress was observed only in the 12-week follow-up, not the 2-week follow-up. The lack of significant difference at 2-week follow-up suggests that it may have been too soon to assess this outcome: the CDEs may have needed more time to integrate their learning into clinical practice. Health professionals have reported elsewhere that it takes time to master new consultation skills, which can hinder post-training implementation [21]. The need for longer follow-up is also a potential explanation as to why no significant changes in frequency of assessing diabetes distress (using a validated questionnaire) were observed in either group. An alternative explanation is that the systemic barriers to implementation (e.g., financial and service-related) were too substantial for the participants to overcome, despite the additional training they received from the e-learning. Of the 18 potential barriers to support for diabetes distress listed in our survey, only ‘Access to screening questionnaires’ was significantly reduced post-intervention at both follow-up timepoints. Notably, both the e-learning and chapter included information about and directed learners to a printable copy of the PAID scale for use in their clinical practice. We were able to further explore the participants’ barriers to implementation of the e-learning qualitatively and will report the findings in a separate publication. Systems thinking approaches have long recognised the impact of physical and social environments upon human behaviour and recommend attention to the underlying structures influencing behaviour [34]. Without addressing the wider context, behavioural changes are unlikely to occur at a population level and may not be sustainable in the long-term [34]. Our e-learning fills a crucial training gap highlighted as a need by CDE’s. But training individuals, while important, will not overcome the systemic barriers raised by the participants as detrimental to providing support for diabetes distress. Our findings provide further evidence that integration of routine support for diabetes distress will require multifaceted approaches and a whole-system shift, backed by policy change and funding support [3].

### Strengths and limitations

A strength of the study is the rigorous development of the active control and intervention learning activities. For the Diabetes Distress chapter of ‘Diabetes and Emotional Health’ (active control activity), this involved formative evaluation comprising literature reviews and several stages of end-user (health professionals) and stakeholder (e.g. academics, health professionals, and people with diabetes) consultation [22]. For ‘Diabetes distress e-learning: A course for diabetes educators’, this included the systematic, evidence-based, and theory-driven intervention mapping approach to development, and multidisciplinary expertise of the development team [26]. The small sample of the current study is larger than the recommended minimum of a pilot study [27], but may not be representative due to self-selection bias (e.g. the sample’s high perceived importance of providing support for diabetes distress may not be congruent with those of the broader CDE community). There was, however, reasonable spread of representation across various workplace and geographical settings, CDE experience, and age. Consistent with broader workforce trends [35], most participants were female. The study is limited by high attrition; of the 76 participants allocated to a learning activity 3% withdrew from the study and 12% did not access their allocated learning activity. Furthermore, of the 65 participants who accessed their learning activity, 51% did not return follow-up data. As such, non-response bias is possible. However, low follow-up response rates are typical in research with health professionals and previous studies exploring non-response biases among health professionals have reported high homogeneity (regarding knowledge, training, attitudes, and behaviour) indicating low risk of non-response bias [28]. The use of study-specific measures may be considered a limitation, however relevant existing validated measures were not available, and they were necessary to measure the specific outcomes of interest of this study. We reiterate that this is a pilot study; the findings, particularly those regarding potential intervention effect, are preliminary and require investigation in a larger trial. It would be valuable if such a trial also included assessment of person-reported outcomes to investigate the indirect impact upon people with diabetes of training health professionals to address diabetes distress.

## Conclusions

This pilot study found the diabetes distress e-learning to be acceptable to CDEs. The study design had reasonable feasibility but requires modification to reduce participant attrition. The e-learning shows potential for improving CDEs’ knowledge, confidence, and behaviours with regard to providing support for diabetes distress. Future larger-scale evaluation of the e-learning is warranted.

## Supplementary Information

**Additional file 1:** **Supplement 1.** Summary of content in ‘Diabetes Distress’ chapter of ‘Diabetes and Emotional Health’.

**Additional file 2:** **Supplement 2.** Reliability analysis: importance and confidence to provide support for diabetes distress.

**Additional file 3:** **Supplement 3.** Participant barriers and enablers to support for diabetes distress: Item endorsements and time-point comparisons (baseline and 2-week follow-up).

**Additional file 4:** **Supplement 4.** Participant barriers and enablers to support for diabetes distress: Item endorsements and time-point comparisons (baseline and 12-week follow-up).

## Data Availability

The datasets generated and/or analysed during the current study are not publicly available as publication of the raw data would not comply with the agreed terms of our ethics approval/participant consent. The datasets are available from the corresponding author on reasonable request.

## Abbreviations

CDE: Credentialled diabetes educator
PAID: Problem Areas In Diabetes scale
BCT: Behaviour change technique

## Footnotes

1 Credentialled Diabetes Educators (CDEs) are health professionals (registered/accredited: nurses, midwifes, dietitians, medical practitioners, pharmacists, podiatrists, exercise physiologists, physiotherapists) who have additionally undertaken a rigorous credentialling program and have specialist knowledge in the field of diabetes education. Credentialling certification is regulated by the Australian Diabetes Educators Association.
