# Supplementary figures and images for: Feasibility and acceptability of e-learning to upskill diabetes educators in supporting people experiencing diabetes distress: a pilot randomised controlled trial
Source: BMC Med Educ. 2022 Nov 9;22:768. doi: 10.1186/s12909-022-03821-w (PMC9644574; doi:10.1186/s12909-022-03821-w)

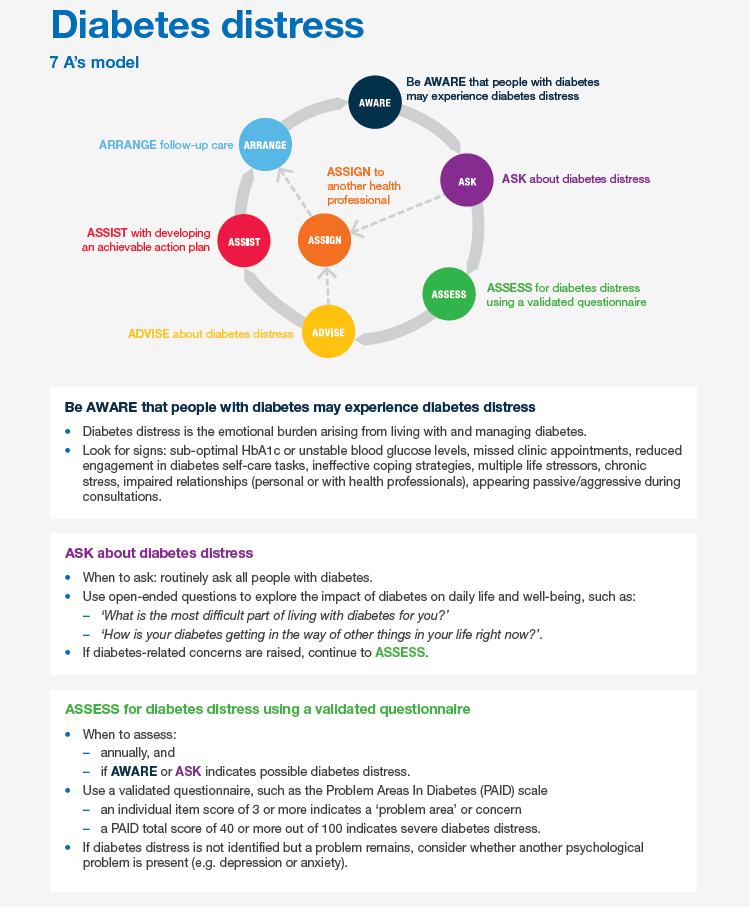


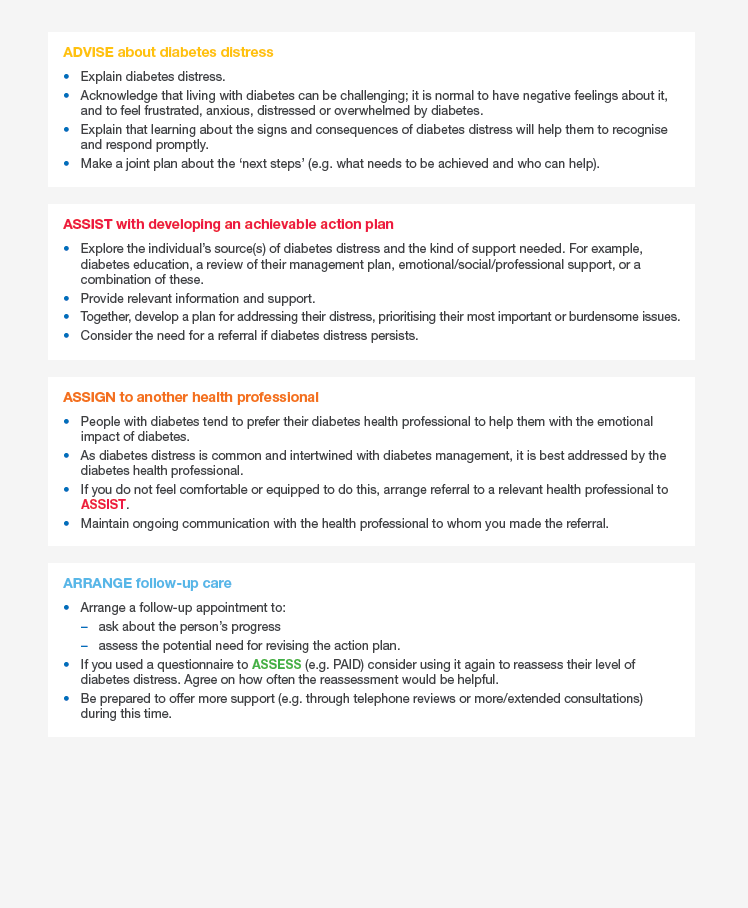

Supplement: Supplementary file 1 — Additional file 1: Supplement 1. Summary of content in ‘Diabetes Distress’ chapter of ‘Diabetes and Emotional Health’. [file 12909_2022_3821_MOESM1_ESM.docx]
