# Supplementary material for: Feasibility and acceptability of e-learning to upskill diabetes educators in supporting people experiencing diabetes distress: a pilot randomised controlled trial
Source: BMC Med Educ. 2022 Nov 9;22:768. doi: 10.1186/s12909-022-03821-w (PMC9644574; doi:10.1186/s12909-022-03821-w)
Supplement: Supplementary file 2 — Additional file 2: Supplement 2. Reliability analysis: importance and confidence to provide support for diabetes distress. [file 12909_2022_3821_MOESM2_ESM.docx]

**Supplement 2: Reliability analysis: importance and confidence to provide support for diabetes distress**

| **Importance** | **Identify** | **In your scope of practice, how important do you believe it is for you to …** | α=0.81 |
| --- | --- | --- | --- |
|  |  | … recognise signs of diabetes distress? |  |
|  |  | … ask open-ended questions to identify diabetes distress? |  |
|  |  | … use a questionnaire to assess diabetes distress (e.g. the PAID or DDS)? |  |
|  |  | … score and interpret a person’s responses to a diabetes distress questionnaire? |  |
|  |  | … talk with a person about their responses to a diabetes distress questionnaire? |  |
|  | **Assist** | **When you have identified that a person has elevated (moderate or severe) diabetes distress, how important do you believe it is for you to …** | α=0.93 |
|  |  | … explore with the person what is causing their diabetes distress? |  |
|  |  | … explore strategies to address diabetes distress with the person? |  |
|  |  | … follow-up with the person about their diabetes distress? |  |
|  | **Refer** | **In your scope of practice, when a mental health referral is required, how important do you believe it is for you to …** | α=0.89 |
|  |  | … talk with the person about their feelings and concerns about mental health referral? |  |
|  |  | … provide suitable mental health referral options to the person? |  |
|  |  | … follow-up with the person about their diabetes distress after the referral? |  |
| **Confidence** | **Identify** | **How confident do you feel to …** | α=0.87 |
|  |  | … recognise signs of diabetes distress? |  |
|  |  | … ask open-ended questions to identify diabetes distress? |  |
|  |  | … use a questionnaire to assess diabetes distress (e.g. the PAID or DDS)? |  |
|  |  | … score and interpret a person’s responses to a diabetes distress questionnaire? |  |
|  |  | … talk with a person about their responses to a diabetes distress questionnaire? |  |
|  | **Assist** | **When you have identified that a person has elevated (moderate or severe) diabetes distress, how confident do you feel to …** | α=0.90 |
|  |  | … explore with the person what is causing their diabetes distress? |  |
|  |  | … explore strategies to address diabetes distress with the person? |  |
|  |  | … follow-up with the person about their diabetes distress? |  |
|  | **Refer** | **When a mental health referral is required, how confident do you feel to …** | α=0.87 |
|  |  | … talk with the person about their feelings and concerns about mental health referral? |  |
|  |  | … provide suitable mental health referral options to the person? |  |
|  |  | … follow-up with the person about their diabetes distress after the referral? |  |

Reliability analysis (Cronbach’s alpha) conducted with baseline sample (N=74)

Response options and scoring: Not at all important/confident (0), Somewhat important/confident (1), Important/confident (2), Very important/confident (3)
