# Supplementary material for: Feasibility and acceptability of e-learning to upskill diabetes educators in supporting people experiencing diabetes distress: a pilot randomised controlled trial
Source: BMC Med Educ. 2022 Nov 9;22:768. doi: 10.1186/s12909-022-03821-w (PMC9644574; doi:10.1186/s12909-022-03821-w)

**Intervention group (N=15)**

**2-week follow-up**

**Baseline**

**Supplement 3: Participant barriers and enablers to support for diabetes distress: Item endorsements and time-point comparisons (baseline and 2-week follow-up)**

Items are presented in the order they appeared in the survey. Item scoring: ‘Hinders me a lot (-2) to ‘Helps me a lot’ (2)

*Significant difference between baseline and 2-week follow-up (Wilcoxon Signed Rank Test): T= 41.00, z=-2.22, r= 0.41, p<0.03

**Significant difference between baseline and 2-week follow-up (Wilcoxon Signed Rank Test): T= 55.00, z=-2.84, r= 0.52, p<0.01

**Baseline**

**2-week follow-up**

N

N

N

N


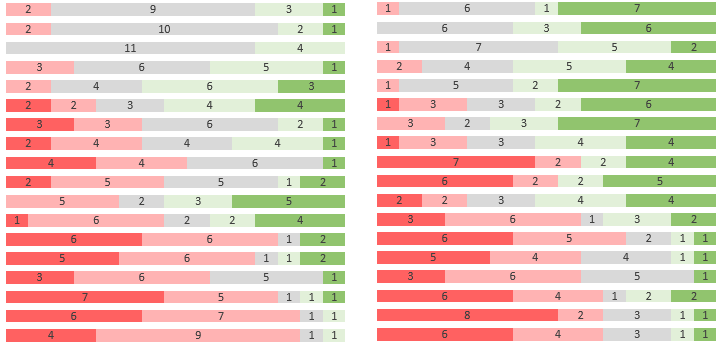


**

*

*

**Active control group (N=18)**


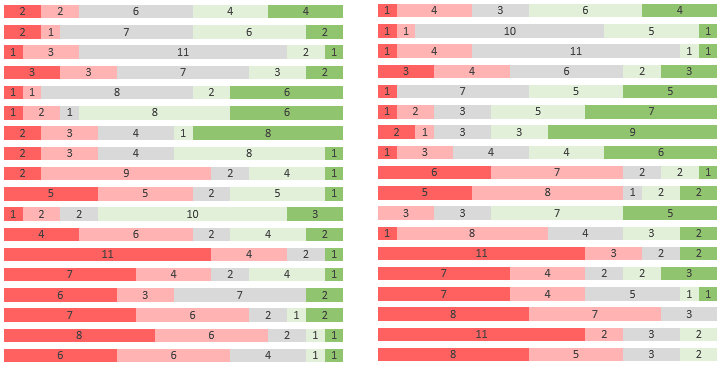


Helps me a little bit

Helps me a lot


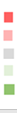


Hinders me a lot

Hinders me a little bit

Neither helps nor hinders me


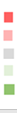

Supplement: Supplementary file 3 — Additional file 3: Supplement 3. Participant barriers and enablers to support for diabetes distress: Item endorsements and time-point comparisons (baseline and 2-week follow-up). [file 12909_2022_3821_MOESM3_ESM.docx]
