# Supplementary material for: Feasibility and acceptability of e-learning to upskill diabetes educators in supporting people experiencing diabetes distress: a pilot randomised controlled trial
Source: BMC Med Educ. 2022 Nov 9;22:768. doi: 10.1186/s12909-022-03821-w (PMC9644574; doi:10.1186/s12909-022-03821-w)
Supplement: Supplementary file 4 — Additional file 4: Supplement 4. Participant barriers and enablers to support for diabetes distress: Item endorsements and time-point comparisons (baseline and 12-week follow-up). [file 12909_2022_3821_MOESM4_ESM.docx]

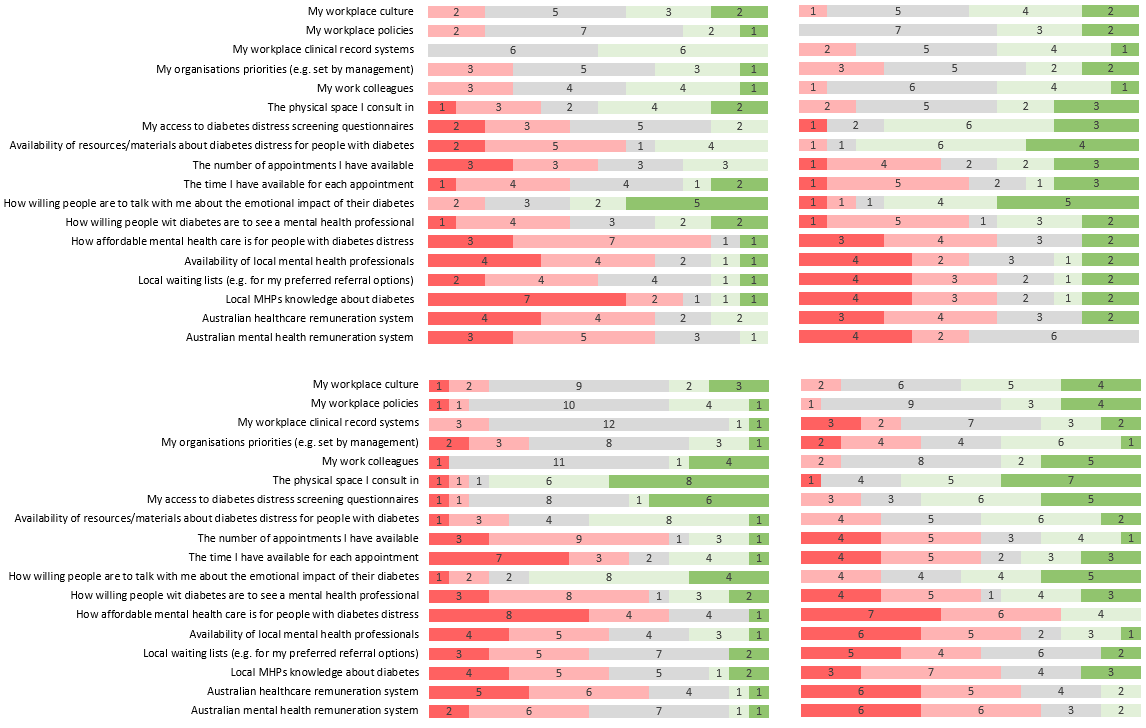


**Baseline**

**12-week follow-up**

N

Items are presented in the order they appeared in the survey. Item scoring: ‘Hinders me a lot’ (-2) to ‘Helps me a lot’ (2)

*Significant difference between baseline and 12-week follow-up (Wilcoxon Signed Rank Test): T= 36.00, z=-2.55, r= 0.52, p<0.01

**Significant difference between baseline and 12-week follow-up (Wilcoxon Signed Rank Test): T= 74.50, z=-2.84, r= 0.58, p<0.01

**Supplement 4: Participant barriers and enablers to support for diabetes distress: Item endorsements and time-point comparisons (baseline and 12-week follow-up)**

N

**

*

**Intervention group (N=12)**

*

**

**Active control group (N=17)**

**12-week follow-up**

**Baseline**


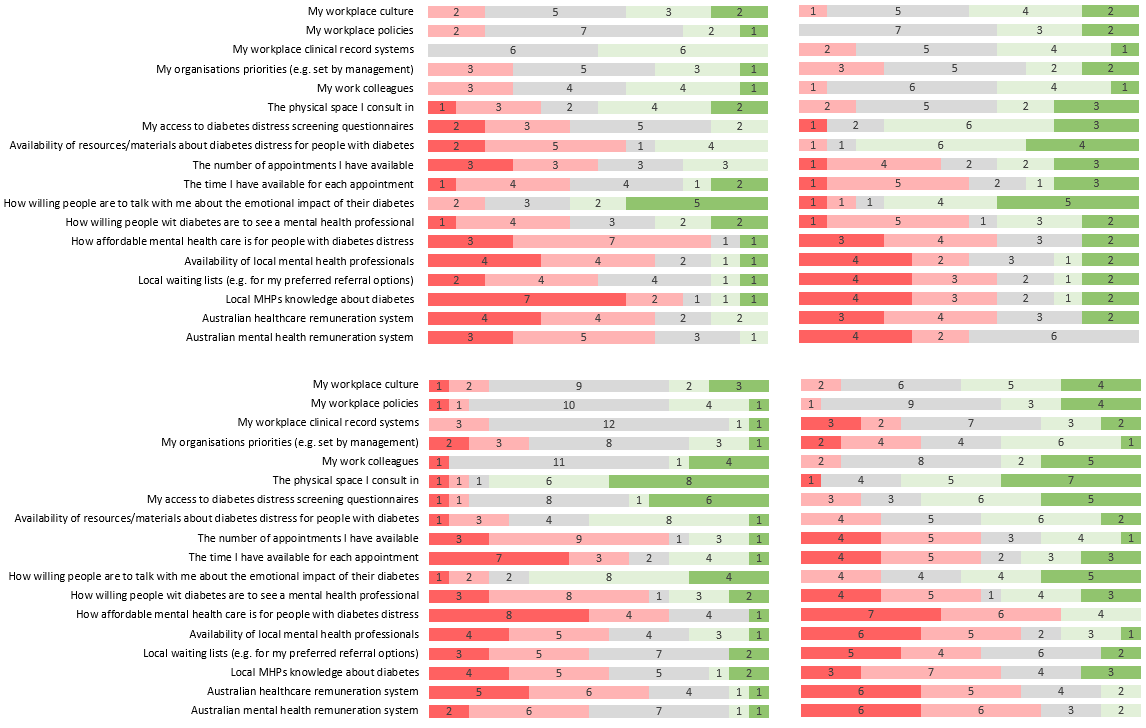


Helps me a little bit

Helps me a lot


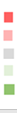


Hinders me a lot

Hinders me a little bit

Neither helps nor hinders me


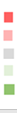


N

N
